# Department of Canine and Feline Medicine and Surgery

**Published:** 1900-11

**Authors:** Cecil French

**Affiliations:** Washington, D. C.


### DEPARTMENT OF CANINE AND FELINE MEDICINE AND SURGERY.

BY CECIL FRENCH, D.V.S.,
WASHINGTON, D. C.

COMPOUND FRACTURE OF THE TIBIA AND FIBULA IN A BLACK-AND-TAN TERRIER; OPERATION; RECOVERY. The subject was a small, dainty specimen of the breed, weighing not more than four or five pounds. When received by me it was walking on three legs, and I was informed that it had sustained injury through falling from a height on to a concrete pavement two days previously.

Examination showed that there existed a complete fracture of the lower extremities of the tibia and fibula of the left leg just above their epiphyses, with over-riding about half an inch in extent. The fractured extremity of the proximal portion of the tibia was displaced inwardly, forming a marked prominence. It had slightly perforated the integument, presumably at the moment of fracture, but was not then visible. The distal portion, which lay on the outer side, was everted and rotated inward.

I endeavored to reduce this fracture by simple methods, but without success, as muscular contraction effectually prevented me from correcting the over-riding. Another attempt with the aid of ether was likewise barren of result.

Nothing now remained but extreme measures. I may state at this juncture that a local veterinarian had previously attempted reduction with the same non-success, and had given the case up, advising that the animal be chloroformed to death.

The operation I am about to describe is exceedingly simple, provided patience, delicacy in technique, and asepticism are observed. It is one that can and should be undertaken in all cases of fracture where, through tendency to over-riding, the broken extremities cannot be maintained in apposition.

The instruments necessary are: scalpel, dissecting forceps, two pairs of hæmostatic forceps, bone-forceps, bone-drill, silver-wire suture, and curved needle. The bone-drill in a case like this can be manufactured out of a lady's ordinary hat-pin by filing and hammering its point to the shape of a spear-blade.

The animal was laid with its back on the table and secured with hopples and quickly placed under the influence of chloroform by means of an inhaler of my own pattern, which I shall describe in the near future. A free incision was made over the prominence —*i. e.*, the inferior extremity of the proximal portion on the inner aspect of the leg, and the edges of the wound were retracted on either side by seizing them with the hæmostatic forceps and allowing the latter to hang over. A little teasing of the deep fascia with the blunt extremity of the scalpel soon exposed the broken surfaces of both portions of the bones. At this stage another attempt at reduction was made, but proved equally futile, and it was necessary to shorten the proximal portion by clipping off a quarter of an inch of bone from the shaft with the bone-forceps. The extremities could then be easily approximated. The fibula, being of slight importance, did not seem to need and did not receive any attention.

In order to maintain the approximated extremities in apposition, suturing with silver wire was next employed. A hole was drilled in each portion, entering at the lateral surface, and emerging on the fractured surface, their exits being made to correspond to one another in position. The wire was introduced, passed through both holes, the parts approximated, the wire twisted, and the ends cut off short and hammered on to the periosteum. A Halstead subcuticular or buried suture of silver wire served to close the wound. A liberal application of xeroform was used and the parts encased in a posterior angular splint well padded with absorbent cotton and held in position with strips of linen made to stick with mucilage. The splint was constructed of one strip of cardboard bent in the centre to form an angle and stitched to retain its shape.

Three days later the wound was examined, and was found to have entirely closed (healing per primam). No stitches needed to be removed. The splint and external dressings were retained for four weeks, and then permanently removed, and at the present day the little dog is in full use of his leg as if nothing had ever happened to it.

Carpipes Unilateralis Congenitalis. In the September number of this volume I described an instance of carpipes (printed carpus by mistake), or club-foot, in the foreleg. The specimen was obtained from the local pound, and during a recent conversation with one of the employés of that institution, I was informed that another dog, similarly affected, had been gathered in and sent to its doom some time previously, as its owner had refused to redeem it by payment of the usual tax. Being interested, I made further inquiries of the owner and the person in whose possession the animal had come into existence, and was informed that "it had been born with its left foot (the same as in the other case) turned up, and had always walked on its knee, and could get about just as well as any other dog."

An Unusual Exhibition of the Maternal Instinct. Immediately following the above description was another headed "The Conditions Reversed," in which I mentioned instances of adoption and nursing between the dog and cat kind, and *vice versa*.

The case I am now about to relate must be considered somewhat remarkable, inasmuch as the animal manifesting the desire for maternal cares was deprived of its ovaries some two years ago. I say deprived of its ovaries guardedly, because it has regularly estruated since the first operation, and on reopening the abdominal cavity recently I found a cyst present at the site of one of the ovaries, which was evidence that a portion of ovarial tissue had been left behind, and consequently, through the function of ovulation still continuing, though only to a modified extent, the animal was still subjective to the maternal instinct. The animal in question is a fox-terrier bitch owned by Mrs. W. A. Little, of Fredericksburg, Virginia.

Ordinarily, it shows extreme aversion to cats. A kitten was introduced into the family, and to the astonishment of every one the terrier was observed to coax it into the basket used as sleeping-quarters. This occurred about two months after the estrual period, which is the time the mammary glands of the non-pregnant bitches often become active, as if in mistaken anticipation of a call to support a family. The kitten quickly learned to suck and thrived well on its diet. The bitch licked it, nursed it tenderly, growled with resentment at the approach of strangers, and when parted later from its adopted offspring grieved with no uncertain grief.

The Dentition of a Mexican Hairless Dog. It is well known that the dentition of the breed of dogs above referred to is usually imperfect, a circumstance not to be wondered at when we remember the common epiblastic origin of the teeth and dermis. Darwin[1] long ago referred to this peculiarity. Waugh[2] examined

[1] The Origin of Species, vol. i. pp. 14, 179.
[2] Journal of Comparative Medicine and Veterinary Archives, 1890, p. 235.

individual members of this breed, and found the tricuspid arrangement of the incisors wanting, absence of the canines, and slower and later development of the molars than in other breeds. Half-breeds had canines in the upper or lower jaw, but not in both jaws.

I have recently had the opportunity to examine the jaws of a five-year-old bitch of pure breed, and found the arrangement of the teeth present as follows: In the upper jaw there were two well-developed incisors, but without any sign of cusps, and one rudimentary one. Two rudimentary canines occupied a normal position. Of the molar group, there was entire absence of true molars, the only representatives being one premolar situated in the position usually occupied by the second premolar, and the fourth premolar, known as the superior sectorial. The anterior one inclined to the rudimentary type, while the sectorial, instead of being composed of two main lobes and a third smaller one, possessed only two lobes, and these were ill-defined. This arrangement was common to both sides.

In the lower jaw there was absolutely no sign of an incisor or a canine. One premolar was present on either side, corresponding in position to the first one of a normal mouth, but still rudimentary in point of development. On either side was a fairly well-developed tooth corresponding in shape to the first true molar or lower sectorial, but occupying a position proper to the last premolar.

These two posterior molars of both jaws not having any teeth to oppose them had caused the formation of indentations in the opposite jaws.

It will thus be seen that not a single tooth possessed by this animal had attained its proper development. I was careful to inquire of the owner and look myself for evidence of premature loss of any of the teeth, but such did not appear to have been the case.

---

*Bulletin No. 25* of the Bureau of Animal Industry contains a report by the Committee on Public Health of the Medical Society of the District of Columbia as to "rabies." It adds to the vast literature already extant upon this subject. If the public could only be encouraged to read literature prepared in bulletins of this kind on diseases such as tuberculosis, the fruits of the work done in controlling that fearful destroyer of human lives would be a greater work than can be hoped for in dealing with so comparatively rare a disease as hydrophobia.

